# Supplementary material for: Data on genetic analysis of atherosclerosis identifies a major susceptibility locus in the major histocompatibility complex of mice
Source: Data Brief. 2016 Nov 19;9:1067–9. doi: 10.1016/j.dib.2016.11.058 (PMC5126130; doi:10.1016/j.dib.2016.11.058)
Supplement: Supplementary file 1 — Supplementary material [file mmc1.docx]

**Conflict of interest**

Authors declared no conflict of interest, including financial, personal or other relationships with other people or organizations, within the past three years that could have inappropriately influenced the work reported here.
