# Supplementary material for: Data on genetic analysis of atherosclerosis identifies a major susceptibility locus in the major histocompatibility complex of mice
Source: Data Brief. 2016 Nov 19;9:1067–9. doi: 10.1016/j.dib.2016.11.058 (PMC5126130; doi:10.1016/j.dib.2016.11.058)
Supplement: Supplementary file 3 — Supplementary material [file mmc3.zip › balb_smj_female_aorta_qtl_dib_shi(1).docx]

*Data article*

**Title:** Quantitative Trait Locus Analysis for Aortic Atherosclerosis in a Female BALB x SM/J Intercross

**Authors:** Andrew T Grainger^1^, Michael B. Jones^2^, Jing Li^2^, Mei-Hua Chen^2^, Ani Manichaikul^3^, Weibin Shi^1,2^

**Affiliations:** ^1^ Departments of Biochemistry & Molecular Genetics and ^2^ Radiology and Medical Imaging, ^3^Public Health Sciences, University of Virginia, Charlottesville, Virginia, USA.

**Contact email:** ws4v@virginia.edu

**Abstract:**

The data presented here are related to the research article, entitled Genetic analysis of atherosclerosis identifies a major susceptibility locus in the major histocompatibility complex of mice, published in Atherosclerosis 2016;254:124 [1]. The supporting materials include original genotypic and phenotypic data obtained from 206 female F2 mice derived from an intercross between BALB and SMJ inbred mice. The F2 mice were fed 12 weeks of Western diet, starting 6 weeks of age. Atherosclerotic lesion size in the aortic root of each mouse is the sum of the top 8 lesion areas. The data is provided in the format required for determining QTLs using two independent programs, J/QTL and PLINK.

**Specifications Table:**

| Subject area | *Genetics* |
| --- | --- |
| More specific subject area | *QTL analysis of atherosclerosis* |
| Type of data | *Tables* |
| How data was acquired | *Aortic lesion size was quantified with AxioVision version 4.8 software.*  *Total and HDL cholesterol, triglyceride, and glucose levels were measured with assay kits*  *Genome-wide genotypic analysis was performed with Illumina LD Linkage Panel.* |
| Data format | *Raw, Analyzed* |
| Experimental factors | *Mice were fed a western diet for 12 weeks.* |
| Experimental features | *Genome-wide genotypic and phenotypic analyses for 206 female F2 mice.* |
| Data source location | *University of Virginia, Charlottesville, Virginia, USA* |
| Data accessibility | *Within this article* |

**Value of the data**

- Providing the raw data for genetic analyses allows for others to have access to the phenotypic quantitative information being addressed and permits them to verify the conclusions reached
- This data is important for researchers that are interested in differences between the phenotypic values of different intercrosses
- Providing raw data is critically important for researchers that are interested in combining datasets for combined cross analysis to look at overlapping QTLs
- The formatting of the data that is put into J/QTL for use in a separate program, PLINK, could be important for future researchers who might want to utilize PLINK for future genetic analyses in other mice intercrosses.

**Data:**

The data shared here is collected for Quantitative Trait Locus (QTL) analysis using 206 female F2 mice derived from an intercross between BALB/cJ and SM/J mice. The data used for J-QTL analysis are presented in Suppl. Table 1. A summary of discovered significant and suggestive QTLs is provided in Table 1.

The file used for PLINK is presented in Suppl. Table 2. The results obtained are present in Table 2.

**Experimental Design, Materials and Methods:**

**Mice**

BALB-Apoe^-/-^ and SM-Apoe^-/-^ mice were generated in our laboratory using the classical congenic breeding strategy [2]. BALB/cJ-Apoe^-/-^ mice were crossed with SM/J-Apoe-/- mice to generate F1s, which were intercrossed by brother-sister mating to generate F2s. Mice were weaned onto a rodent chow diet at 3 weeks of age. At 6 weeks of age, female F2 mice were started with a Western diet containing 21% fat, 34.1% sucrose, 0.15% cholesterol, and 19.5% casein and maintained on the diet for 12 weeks.

**Quantitation of aortic atherosclerosis**

The vasculature of mice was perfusion-fixed with 4% PFA (paraformaldehyde) through the left ventricle of the heart. The aortic root and adjacent heart were harvested, embedded in optimal cutting temperature compound and cross-sectioned in 10-μm thickness. Sections were stained with oil red O and hematoxylin and counterstained with fast green. Atherosclerotic lesion areas were measured using Zeiss AxioVision 4.8 software. The eight largest aortic lesion areas were added up for each mouse and this sum was used for statistical analysis [3].

**Genotyping**

The Illumina LD linkage panel consisting of 377 SNP loci was used to genotype the F2 cohort. Microsatellite markers were typed for chromosome 8 where SNP markers were uninformative in distinguishing the parental origin of alleles. DNA samples from the two parental strains and their F1s served as controls. Uninformative SNPs were excluded from QTL analysis. SNP markers were also filtered based on the expected pattern in the control samples, and F2 mice were filtered based on 95% call rates in genotype calls. After filtration, 149 markers were included in genome-wide QTL analysis.

**Acknowledgements:** This work was supported by NIH grants DK097120 and HL112281.

*References*

*[1] A.T. Grainger, M.B. Jones, J. Li, M.H. Chen, A. Manichaikul, W. Shi, Genetic analysis of atherosclerosis identifies a major susceptibility locus in the major histocompatibility complex of mice, Atherosclerosis****254****(2016) pp. 124-32.*

*[2] S. Liu, J. Li, M.H. Chen, Z. Liu, W. Shi, Variation in Type 2 Diabetes-Related Phenotypes among Apolipoprotein E-Deficient Mouse Strains, PLoS One****10****(2015) pp. e0120935.*

*[3] A.T. Grainger, M.B. Jones, J. Li, M.H. Chen, A. Manichaikul, W. Shi, Genetic analysis of atherosclerosis identifies a major susceptibility locus in the major histocompatibility complex of mice, Atherosclerosis****254****(2016) pp. 124-32.*
